# Primary Extra-Osseous Ewing Sarcoma of the Thyroid: A Case Report and Review of the Literature

**DOI:** 10.1007/s12105-021-01365-x

**Published:** 2021-07-26

**Authors:** Amanda H. Seipel, Hiba Mechahougui, Nicolas Mach, Frédéric Triponez, William C. Faquin, Claudio De Vito

**Affiliations:** 1 Department of Clinical Pathology, Geneva University Hospital, Geneva, Switzerland; 2 Department of Clinical Oncology, Geneva University Hospital, Geneva, Switzerland; 3 Department of Thoracic and Endocrine Surgery, Geneva University Hospitals and Faculty of Medicine, University of Geneva, Geneva, Switzerland; 4 Department of Pathology, Massachusetts General Hospital and Harvard Medical School, Boston, MA 02114 USA

**Keywords:** Thyroid gland, Extra-osseous Ewing sarcoma, Cyto-histological correlation, Molecular analysis

## Abstract

Extra-osseous Ewing sarcoma (ES) is a rare and aggressive malignant tumor found in a variety of organs. Primary ES of the thyroid is exceedingly rare and few cases have been documented to date. We describe the case of a 54-year old woman with a history of breast carcinoma in whom a unique hypermetabolic left thyroid nodule was identified during a follow-up PET-CT scan. An ultrasound examination showed a hypoechogenic nodule of 3.7 cm. A cytological diagnosis of poorly differentiated thyroid carcinoma was made, and a total thyroidectomy was performed. The surgical specimen revealed a poorly differentiated neoplasm composed of medium-sized cells with scant cytoplasm, expressing pancytokeratin, CD99 and NKX2.2 but lacking p63 and p40 expression. Molecular analysis revealed a *EWSR1-FLI1* fusion transcript supporting the diagnosis of a primary extra-osseous ES of the thyroid. The patient received adjuvant chemotherapy and has no evidence of recurrent disease.

## Introduction

Thyroid cancers encompass a wide range of tumors, from indolent cases with low mortality to extremely aggressive malignancies with short overall survival. Proper diagnostic work-up and classification are crucial to tailor patient treatment. Well-differentiated thyroid carcinoma such as papillary, follicular and Hürthle cell carcinoma account for more than 95% of all cases, while other subtypes are less frequently seen [1]. Mesenchymal thyroid neoplasms are exceedingly rare among which Ewing sarcoma (ES) represents only 2% [2]. ES is a member of the so-called “small round blue cell” tumors, most commonly arising in bone and most frequently found in children and adolescents. Extra-osseous Ewing sarcoma (EES) is less frequent and can occur at a wide variety of anatomic [3]. Several recent reports have described an adamantinoma-like Ewing sarcoma (ALES) arising in the thyroid gland, based on the identification of *EWSR1* rearrangement in neoplasms showing the classic ES morphology with epithelial differentiation (keratin and p63 or p40 expression) [4–8], and carcinoma of the thyroid with Ewing family tumor elements (CEFTEs) when associated with papillary thyroid carcinoma [9–12]. Here we present the case of a patient diagnosed with EES located in the left thyroid lobe and a review of the current literature.

## Case

### Clinical History

A 54-year old woman presented with a 3.7 cm nodule in the left thyroid lobe during a routine PET-scan follow-up for a triple negative breast carcinoma, without BRCA-1 or BRCA-2 mutation, treated with neoadjuvant chemotherapy, surgery, and radiotherapy. Physical examination revealed an asymmetric thyroid gland with a firm nodule of the left thyroid lobe, without any palpable lymphadenopathy. Ultrasound imaging revealed a hypoechogenic and hypervascularized nodule measuring 3.7 × 3.1 × 2.1 cm without calcifications which was classified radiologically as TiRADS 4.

**Fig. 1:**
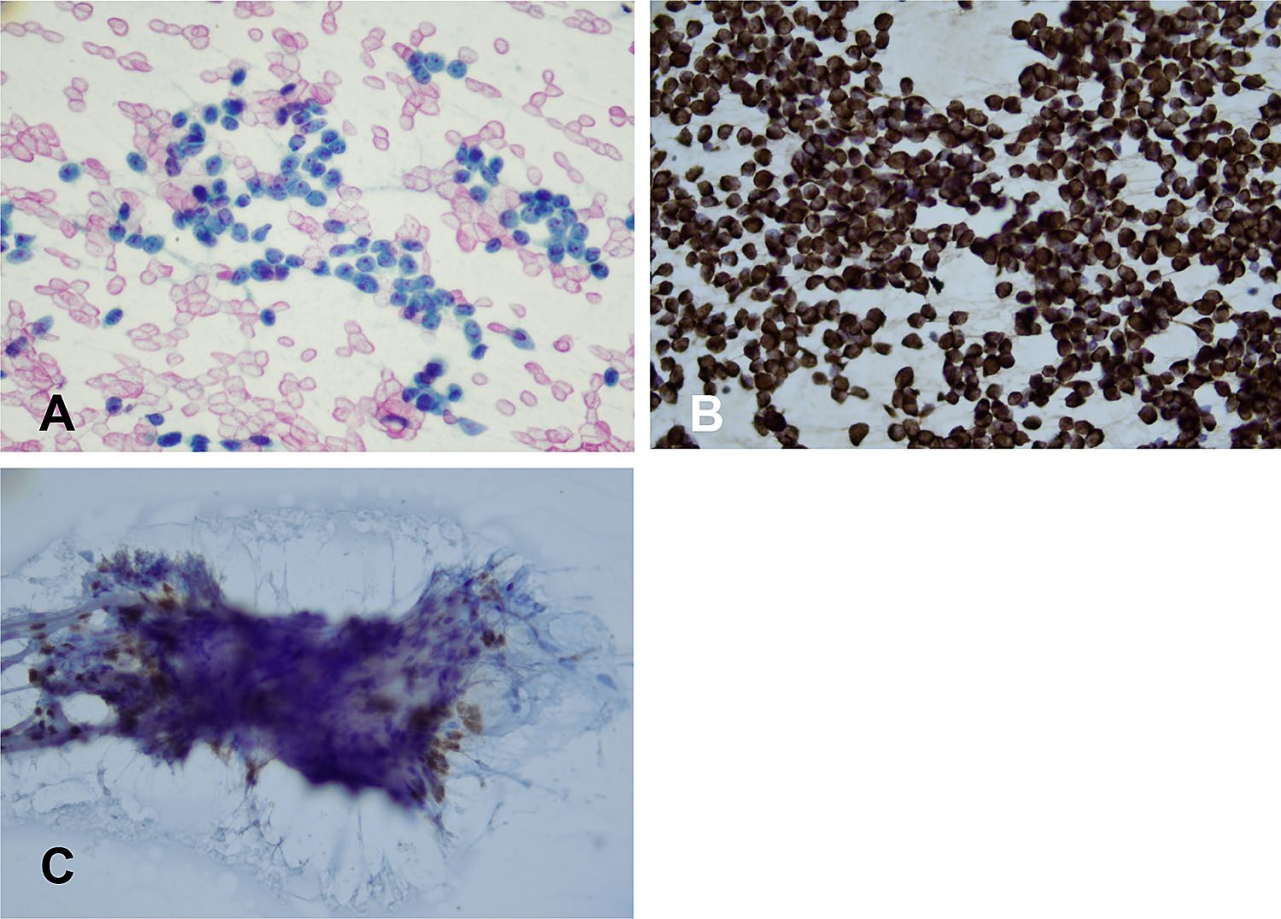
Cytological findings in the FNA. **A** Monomorphic proliferation of dyscohesive cells, with scant cytoplasm and visible nucleoli, PAP stain (× 100). **B** Keratin immunocytochemistry (× 100). **C** TTF1 immunocytochemistry showing nuclear expression in a subset of cells (× 100)

An US-guided fine needle aspiration (FNA) was performed which showed a monomorphic proliferation of dyscohesive cells isolated and in groups, with scant cytoplasm and distinct small nucleoli (Fig. 1A). The cells stained diffusely for pancytokeratin (Fig. 1B), and scattered cells stained for TTF-1 (Fig. 1C) and thyroglobulin (data not shown). GATA-3, calcitonin, mammaglobin, estrogen and progesterone receptors were negative (data not shown). Based on the cytomorphology and immunocytochemical profile a diagnosis of poorly differentiated thyroid carcinoma (PDTC) was made, and the patient underwent a total thyroidectomy.

The surgical specimen of the left thyroid lobe showed a poorly delineated cream-colored nodule with hemorrhagic zones, measuring 4 cm, occupying the superior and medial part of the thyroid, which was extensively sampled. The right lobe contained a small, sclerotic nodule.

**Fig. 2:**
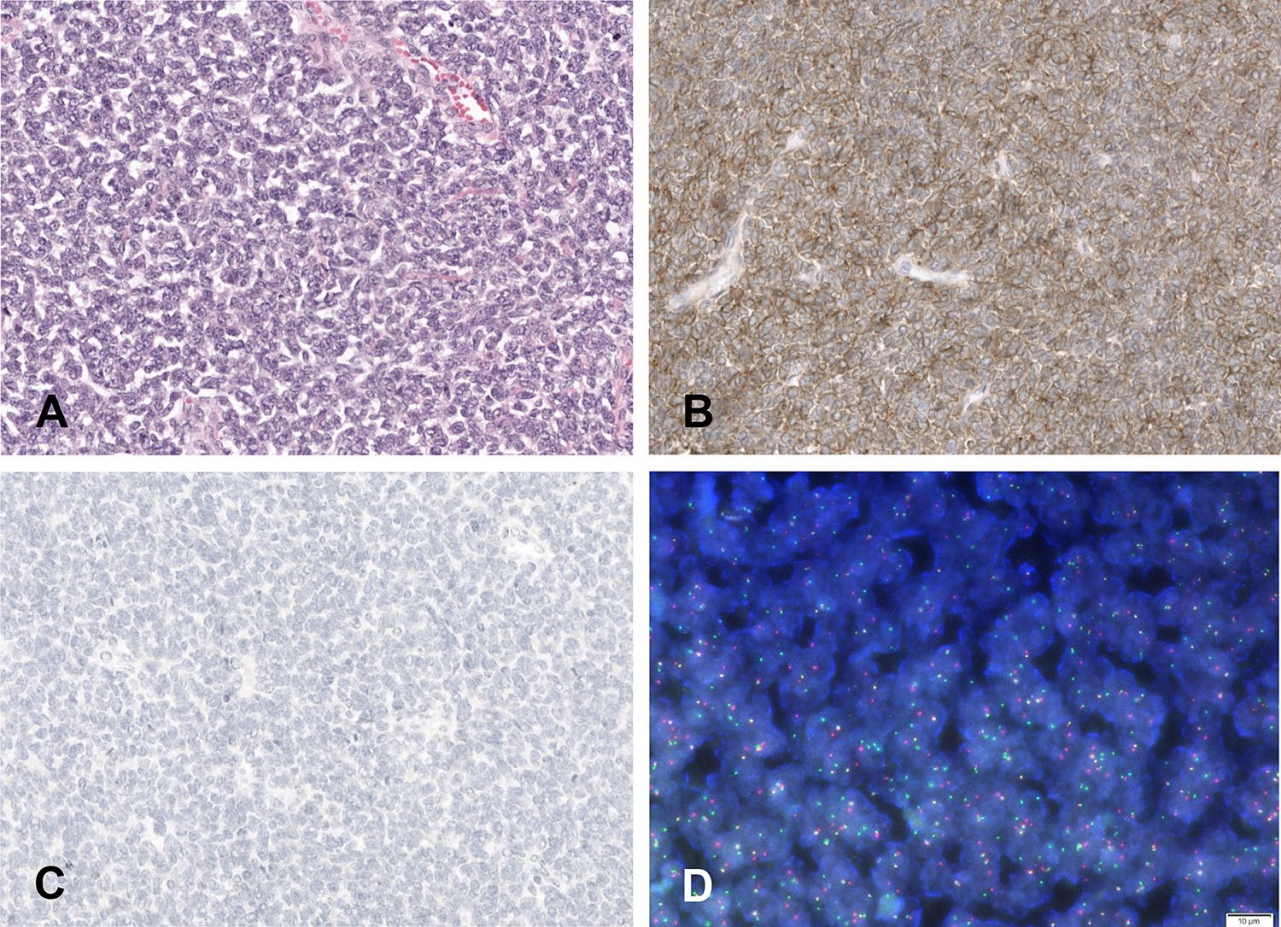
Histological, immunohistochemical and FISH findings in the surgical specimen. **A** Neoplasm composed of small to medium sized cells with scant cytoplasm and enlarged nucleus with evenly distributed chromatin and inconspicuous nucleoli (H&E*). **B** Diffuse CD99 expression (× 100). **C** Absence of p40 expression (× 100), **D** FISH analysis showing *EWSR1* gene rearrangement using a break apart probe. ^*^Hematoxylin and eosin

Histological analysis showed a solid cellular proliferation separated by fibrous bands, composed of small to medium sized cells with scant cytoplasm and an enlarged nucleus. Chromatin was evenly distributed and nucleoli were inconspicuous (Fig. 2A). Mitoses were scarce. Multifocal vascular invasion was observed, without any evidence of lymphatic or perineural invasion. The tumor was limited to the thyroid gland but focally in contact with the surgical margins. Immunohistochemistry showed diffuse expression for pancytokeratin, epithelial membrane antigen (EMA), CD117, CD99 (Fig. 2B) and NKX2.2. The cells were negative for PAX-8, thyroglobulin, CEA, calcitonin, p63, p40 (Fig. 2C), chromogranin, synaptophysin, hormonal receptors (ER, PR), GATA3, mammaglobin and BRST2. P53 showed a wild type expression. INI-1 and BRG-1 expression was conserved. Scattered non-neoplastic thyrocytes expressing TTF-1 were observed entrapped within the tumor. The surrounding thyroid parenchyma showed mild chronic thyroiditis.

Based on the histomorphology and immunohistochemical profile, an RNA-based fusion panel (Illumina TruSight Fusion panel, Illumina, San Diego, CA) was performed and identified an *EWSR1-FLI1* fusion transcript, validated by a *EWSR1* FISH break apart probe (Fig. 2D) supporting the diagnosis of EES. An additional custom NGS targeted panel (including more than 400 genes) did not identify any gene mutation. Since PET/CT did not reveal involvement of other anatomic sites, a diagnosis a primary EES of the thyroid gland was made. Based upon the overall pathologic and clinical findings, the multidisciplinary institutional tumor board panel recommended adjuvant chemotherapy.

The patient then received four cycles of adjuvant polychemotherapy with VIDE (Vincristine, Ifosfamide, Doxorubicin, Etoposide) and Peg G-CSF.

Adequate cardiac function was confirmed by cardiac ultrasound as part of the pre-treatment protocol and because the patient had already received 360 mg/m^2^ of Epirubicine in 2016 for the multimodality treatment of a triple negative breast cancer. Chemotherapy was complicated by grade 3 asthenia, and grade 4 neutropenia, anemia, and thrombocytopenia requiring a 20% dose reduction for the last two cycles. Treatment was completed with adjuvant radiotherapy (total dose 45 Gy delivered in 25 sessions). As this patient presented with two rare and aggressive neoplasms, triple negative breast cancers and a sarcoma developed within the field of her breast cancer radiotherapy, genetic predisposition such as Li-Fraumeni syndrome was suspected. Genetic analysis of eight genes: *TP53, PTEN, STK11, PALB2, ATM, CHEK 2,* did not reveal any anomaly.

Fifteen months after surgery, the patient is in remission with no local recurrence no distant metastases.

## Discussion

Primary ES family tumors tend to occur most commonly in bone, primarily in adolescents and young adults, and is characterized by chromosomal rearrangements involving the *EWSR1* gene [3]. More recently EES termed ALES has been increasingly recognized in the head and neck region including salivary gland and thyroid [13], and also carcinoma of the thyroid with CEFTEs [9]. Although ES can express epithelial markers such as pankeratin [14], p63 and/or p40 were reported only in ALES [13]. In our case, the absence of p40 and p63 expression and the lack of papillary thyroid carcinoma foci associated with the tumor support the classification of EES over ALES or CEFTE.

The pre-operative FNA diagnosis remains challenging (Table 1). As reported by Kabata et al. [15], a correct FNA diagnosis was not achieved in any case. More recently one report [16] describes a successful interpretation by FNA. It is interesting to note that the few cases that were able to correctly diagnose the tumor were done in association with a core needle biopsy [15, 16] or excision biopsy [4] (Table 1).

As in our case, a potential pitfall in the interpretation of cytological specimens is the diffuse expression of keratin, which may lead to the misclassification of the cancer as PDTC. This is especially true when entrapped benign, TTF-1 positive thyrocytes are present in the FNA sample as encountered in our case, and similarly as described by Chikeka et al. [17], or when p40 or p63 are only expressed focally [6, 8], or are absent.

The differential diagnosis encompasses a broad range of neoplasms including metastatic basaloid squamous cell carcinoma, which expresses keratin, p63 and p40, and medullary thyroid carcinoma which can be confirmed by testing for calcitonin and CEA expression. In the setting of a calcitonin-negative tumor, other neuroendocrine entities of the thyroid gland including intrathyroidal paraganglioma should be considered. Intrathyroidal thymic carcinoma based on the immunohistochemical profile (positivity for keratin, EMA and CD117) should also be excluded.

Given the high-grade nature of the tumor and unusual immunoprofile and histomorphology, a thyroid gland metastasis should also be considered. In our case, a metastasis from the patient’s known breast cancer was excluded using immunohistochemical markers for GATA-3, mammaglobin, and BRST2. This clinical presentation of EES is reminiscent of the case reported by Maldi et al. [6].

ES is an aggressive disease with a 70% overall 5-year relative survival rate [3]. However, the difference in prognosis between EES and ES is still controversial. A report from the Children’s Oncology Group analyzing a cohort of two clinical trials including 213 extraskeletal ES and 826 skeletal ES showed that EES were associated with a slightly better event-free survival [18]. In contrast, Lynch et al. [19] did not find any impact on survival in the adult population. Most patients with thyroid involvement presented with angio-lymphatic invasion and a small subset with lymph node metastasis (Table 1). The majority of patients received adjuvant chemotherapy and all showed no evidence of disease in the clinical follow-up except for one patient [20]. However, it is important to emphasize that the clinical follow-up for most patients was limited, and therefore any conclusions about the long term patient prognosis is uncertain.

**Table 1:** Summary of the clinico-pathological features of the reported cases of extra-osseous Ewing sarcoma, adamantinoma-like Ewing sarcoma and carcinomas of the thyroid with Ewing family tumor elements

	Age	Sex	FNA/ core needle biopsy	Tumor size	Adverse factor	Treatment	Follow up/outcome	p40/p63 expression	other
Adapa et al. [4]	9	F	Ewing sarcoma	6.0 cm	Lymph node metastasis	Chemotherapy, surgery, radiotherapy	6 years, ANED	N/A	
Eloy et al. [11]	42	F	Follicular tumor	2.5 cm	Vascular invasion	Total thyroidectomy	38 months, ANED	Positive	CEFTE
Maldi et al. [6]	66	M	Malignant tumor of possible neuroectodermal origin	4.5 cm	Vascular invasion	Total thyroidectomy, no chemotherapy or radiotherapy	8 months/ AWED	Focal expression	
Chirila et al. [7]	48	M	Giant B-cell non-Hodgkin lymphoma	10 cm	Positive surgical margins	CHOP (initial diagnosis of B cell non Hodgkin lymphoma), followed by etoposide and carboplatin	1 month, died from brain metastasis	N/A	No validation of EWSR1 fusion
Chan et al. [5]	23	M	Not performed	4.7 cm	Vascular invasion, lymph node metastasis	Total thyroidectomy, vincristine, actinomycin D, cyclophosphamide etoposide and radiotherapy	under chemotherapy	N/A	
Chan et al. [5]	67	F	Bethesda IV	4.0 cm	Vascular invasion	Total thyroidectomy (2 steps), chemotherapy	22 months ANED	N/A	
Eloy et al. [12]	24	M	Not performed	5.3 cm	Vascular invasion	Total thyroidectomy (2 steps), radioactive iodine	13 years/ ANED	Positive	CEFTE
Bishop et al. [8]	19	M	N/A	N/A	N/A	Surgery, additional therapy pending	0 month	Focally positive (< 5%)	
Bishop et al. [8]	36	F	N/A	N/A	N/A	Surgery, additional therapy pending	0 month	Positive	
Kabata et al. [15]	34	M	Small-cell malignant neoplasm, most likely of hematologic origin/ core needle bx: ewing sacoma	6.0 cm	Not reported	Neo-adj. chemotherapy isthmolobectomy, cervical lymphadenectomy, radiotherapy (discontinued)	18 months	N/A	
Ongkeko et al. [20]	36	M	Suggestive of malignancy	7.0 cm	Extrathyroidal extension, lymphovascular invasion, lymph node metastases	Total thyroidectomy, medial and right lateral neck dissection, chemotherapy	2 years, ANED pancreatic metastasis treated by chemotherapy	N/A	
Morlote et al. [21]	20	F	Suspicious for carcinoma	4.0 cm	Not reported	Total thyroidectomy, chemotherapy, radiotherapy	7 months ANED	Positive	
Jones and Maleki [16]	21	M (transgender)	ALES	7.0 cm	N/A	N/A	N/A (numerous lytic bone lesions)	Positive	
Chikeka et al. [17]	15	F	Bethesda VI	3.0 cm	Positive surgical margins	Total thyroidectomy, chemotherapy, radiotherapy	N/A	Positive	
Taccogna et al. [10]	40	M	Bethesda V	6.5 cm	Not reported	Total thyroidectomy	6 months ANED	Positive	CEFTE
Current case	54	F	Bethesda VI PDTC	4.0 cm	Vascular invasion, positive surgical margins	Total thyroidectomy, chemotherapy, radiotherapy	15 months ANED	Negative	

*ANED* alive, no evidence of disease, *AWED* alive with evidence of disease, *N/A* not available, *CEFTE* carcinoma of the thyroid with Ewing family tumor elements, *ALES* adamantinoma like Ewing sarcoma

In conclusion, correct pre-operative diagnosis of extra-osseous ES remains challenging. Pathologists, including those specializing in cytopathology, should be aware that ES should be considered in the differential diagnosis of poorly differentiated tumors of the thyroid, including those with diffuse keratin expression and focal expression of p40/p63. For such cases, CD99 and NKX2.2 immunostaining or molecular analysis with NGS or FISH can be helpful to properly classify the tumor which is of crucial importance for proper patient management.

## Data Availability

Not applicable.
